# Water Structure Recovery in Chaotropic Anion Recognition: High-Affinity Binding of Dodecaborate Clusters to γ-Cyclodextrin**

**DOI:** 10.1002/anie.201412485

**Published:** 2015-05-07

**Authors:** Khaleel I Assaf, Merve S Ural, Fangfang Pan, Tony Georgiev, Svetlana Simova, Kari Rissanen, Detlef Gabel, Werner M Nau

**Affiliations:** Department of Life Sciences and Chemistry, Jacobs University BremenCampus Ring 1, 28759 Bremen (Germany); University of Jyvaskyla, Department of Chemistry, Nanoscience CenterP.O. Box. 35, 40014 University of Jyvaskyla (Finland); Institute of Organic Chemistry with Center of PhytochemistryBulgarian Academy of Science, 1113 Sofia (Bulgaria)

**Keywords:** boron clusters, cyclodextrins, Hofmeister series, host–guest complexes, supramolecular chemistry

## Abstract

Dodecaborate anions of the type B_12_X_12_^2−^ and B_12_X_11_Y^2−^ (X=H, Cl, Br, I and Y=OH, SH, NH_3_^+^, NR_3_^+^) form strong (K_a_ up to 10^6^ L mol^−1^, for B_12_Br_12_^2−^) inclusion complexes with γ-cyclodextrin (γ-CD). The micromolar affinities reached are the highest known for this native CD. The complexation exhibits highly negative enthalpies (up to −25 kcal mol^−1^) and entropies (TΔS up to −18.4 kcal mol^−1^, both for B_12_I_12_^2−^), which position these guests at the bottom end of the well-known enthalpy-entropy correlation for CDs. The high driving force can be traced back to a chaotropic effect, according to which chaotropic anions have an intrinsic affinity to hydrophobic cavities in aqueous solution. In line with this argument, salting-in effects revealed dodecaborates as superchaotropic dianions.

Association phenomena in aqueous solution, whether between a macrocyclic host and an encapsulated guest or between a biological receptor and its corresponding substrate, are frequently accounted for in terms of a conglomerate driving force, the hydrophobic effect. Regardless of the precise description of the contributors to the hydrophobic effect,[1] it is intuitive that the tendency of a suitably sized guest molecule or residue to become encapsulated inside a hydrophobic macrocyclic cavity scales with its own hydrophobicity, which in turn relates inversely to its water solubility. Exceptionally large affinities (picomolar and below for cucurbiturils as hosts) can thus be reached for highly hydrophobic adamantane, diamantane, or triamantane residues as guests.[2] We now report that highly water-soluble dianionic dodecaborates can form surprisingly strong inclusion complexes with macrocyclic hosts, γ-cyclodextrin in particular. We hold a hitherto underestimated driving force, the “chaotropic effect”, responsible for this affinity.

Borate clusters of the types B_12_X_12_^2−^ and B_12_X_11_Y^2−^ (X=H, Cl, Br, I and Y=OH, SH, NH_3_^+^, NR_3_^+^; Figure 1) are poorly coordinating and weakly basic inorganic anions with icosahedral structure and a permanent double negative charge of the core.[3] Their discovery in the 1960s led to numerous applications in medicinal chemistry and materials science,[4] among which their use in neutron capture therapy of cancer stands out as a practically relevant one.[5] The host–guest chemistry of these hydrophilic cluster anions has not been previously described.[6]

**Figure 1:**
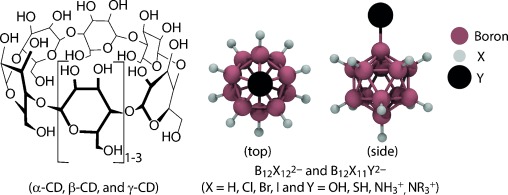
Molecular structure of cyclodextrins (left) and top as well as side views of dodecaborate clusters (right).

Cyclodextrins (CDs, Figure 1) are well-known for encapsulating a wide range of hydrophobic organic[7] and organometallic compounds in their cavity,[8] with few examples of inorganic guests.[9] The complexation of dodecaborates with different CD homologues and derivatives was investigated by ^1^H NMR spectroscopy,[10] which was made possible by their high solubility (for example, 50 mm for Na_2_B_12_I_12_ and more than 3 m for Na_2_B_12_H_11_SH). ^1^H NMR titrations were conducted for all clusters (see the Supporting Information); the largest spectral changes were observed for γ-CD as host. In particular, we witnessed a pronounced complexation-induced shift of the H3 proton (Figure 2 a,b), which is located inside the cavity near the secondary hydroxyl rim, signaling the formation of an inclusion complex. Some clusters (B_12_H_11_SH^2−^, see the Supporting Information) caused not only a significant down-field shift for H3 but an even larger one for H5 (for example, 0.17 versus 0.09 ppm), which confirmed that the dianions protruded deeply into the hydrophobic cavity (Figure 2 c). For the B_12_H_11_NR_3_^−^ clusters (with R=Me, Et, *n*Pr, *n*Bu), we observed selective 2D-ROESY cross-peaks between the aliphatic protons and the H-3 proton of γ-CD, that is, the functional groups Y are positioned near the wider rim (see the Supporting Information).

**Figure 2:**
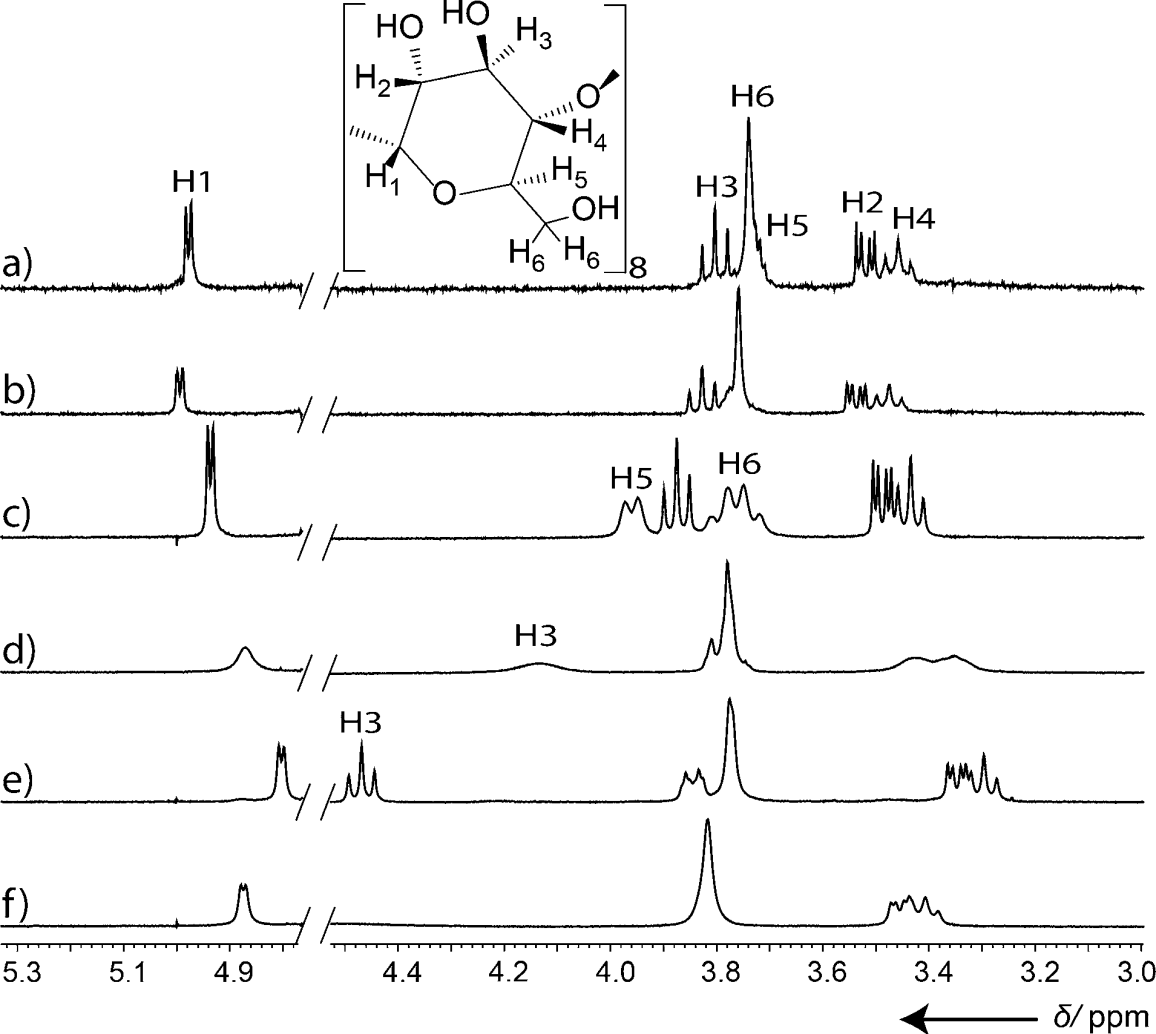
^1^H NMR spectra of a) free γ-CD and its complexes with b) B_12_H_12_^2−^, c) B_12_H_11_SH^2−^, d) B_12_Cl_12_^2−^, e) B_12_Br_12_^2−^, and f) B_12_I_12_^2−^, all as sodium salts.

The NMR titration data for B_12_H_12_^2−^ and the clusters of the type B_12_H_11_Y^2−^ (see the Supporting Information) could be well-fitted according to a 1:1 complexation model, also confirmed by Job plot analysis (Supporting Information). The resulting association constants for γ-CD are shown in Table 1. The guest affinity trends can be largely rationalized in terms of established arguments from the toolbox of host–guest chemistry. For example, with reference to the parent, B_12_H_12_^2−^, the more lipophilic SH substituent increases the affinity, while the more hydrophilic OH group decreases the affinity, which establishes the range of the respectable association constants (0.62–7.8×10^3^ L mol^−1^) for the non-halogenated clusters.

**Table 1:** Association constants *K*_a_ of dodecaborate cluster anions with γ-CD and associated thermodynamic parameters (in kcal mol^−1^).

Borate cluster[a]	*K*_a_ [10^3^ L mol^−1^]	Δ*H*°	*T*Δ*S*°	Δ*G*°
B_12_H_11_OH^2−^	0.62[b]			
B_12_H_11_N(*n*Pr)_3_^−[d]^	1.1[b]			
B_12_H_11_NH_3_^−^	1.7[b]			
B_12_H_12_^2−^	2.0[b]			
B_12_H_11_SH^2−^	7.8[b], 9.2[c]	−5.7	−0.3	−5.4
B_12_Cl_12_^2−^	17[c]	−14.4	−8.6	−5.8
B_12_Br_12_^2−^	960[c]	−21.4	−13.3	−8.1
B_12_I_12_^2−^	67[c]	−25.0	−18.4	−6.6
B_12_I_11_NH_3_^−^	25[c]			

[a] Measured as sodium salts at 25 °C for a 1:1 complexation model.

[b] ^1^H NMR titration in D_2_O.

[c] Measured by ITC in neat water. [d] Potassium salt.

Upon complexation of the perhalogenated dodecaborates (B_12_X_12_^2−^ with X=Cl, Br, I) by γ-CD, the ^1^H NMR spectra showed large down-field shifts for the inner H3 and H5 protons (Figure 2 d–f and the Supporting Information) and up-field shifts of the outer H1, H2, and H4 ones, all indicative of deep inclusion. The shift was largest for H5 in the γ-CD⋅B_12_Br_12_^2−^ complex (0.7 ppm). All perhalogenated clusters showed very strong binding to γ-CD, such that we needed to resort to isothermal titration calorimetry (ITC) to determine precise binding constants. The highest affinity was obtained for B_12_Br_12_^2−^ (9.6×10^5^ L mol^−1^, Figure 4 a), followed by B_12_I_12_^2−^ (6.7×10^4^ L mol^−1^) and B_12_Cl_12_^2−^ (1.7×10^4^ L mol^−1^). This up-and-down trend with increasing cluster size pointed to an ideal size matching for the intermediary brominated cluster (see the Supporting Information).

We obtained single crystals from γ-CD/B_12_Br_12_^2−^ mixtures and solved the interesting XRD structure (Figure 3 a).[11] The CDs pack in the unit cell forming a formal tubular crystal lattice (see the Supporting Information). Two γ-CDs were observed to cap a dodecaborate cluster tightly (Br=H=C distances ca. 3 Å), while the two wider CD rims were held together by intermolecular hydrogen bonds. It should be noted that although the complexation stoichiometry in the solid phase (2:1) differs from that established in aqueous solution (predominantly 1:1, as established by the Job plot and ITC titrations; see the Supporting Information), the tendency for deep immersion is reflected in both phases.

**Figure 3:**
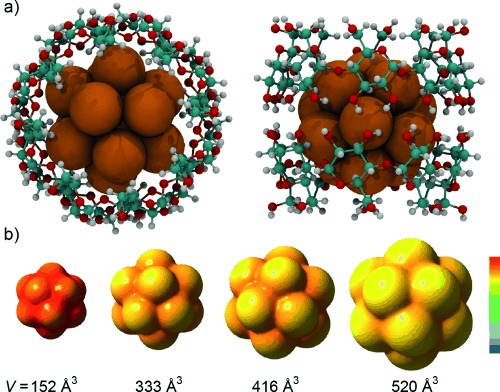
a) Top (left) and side (right) views of the XRD structures of the inclusion entrapment of the B_12_Br_12_^2−^ cluster into the γ-CD dimer. For the sake of clarity, the severely disordered B_12_Br_12_^2−^ cluster is visualized by an ideal, but XRD-based, B_12_Br_12_^2−^ cluster (see the Supporting Information). b) Size comparison and DFT-computed electrostatic potential maps for B_12_H_12_^2−^, B_12_Cl_12_^2−^, B_12_Br_12_^2−^, and B_12_I_12_^2−^; the red to blue surface color range spans −180.0 to +180.0 kcal mol^−1^.

For the highest-affinity clusters, ITC was used to analyze the complexation thermodynamics (Table 1). Invariably, the binding is an enthalpically driven process. There is a good correlation between enthalpy and guest size: the enthalpy of complexation (Δ*H*°) increases from B_12_H_11_SH^2−^ to B_12_I_12_^2−^, in line with increasing dispersion interactions. This trend is counterbalanced by an increasing entropic penalty, that is, enthalpy-entropy compensation applies (Figure 4 b), as is common for CDs.[7] Noteworthy, however, the binding of the highest-affinity perhalogenated clusters stands out in this correlation owing to their very exothermic binding and associated large entropic penalty, which exceed, for the B_12_I_12_^2−^ cluster, the values known for any native CD complex.

**Figure 4:**
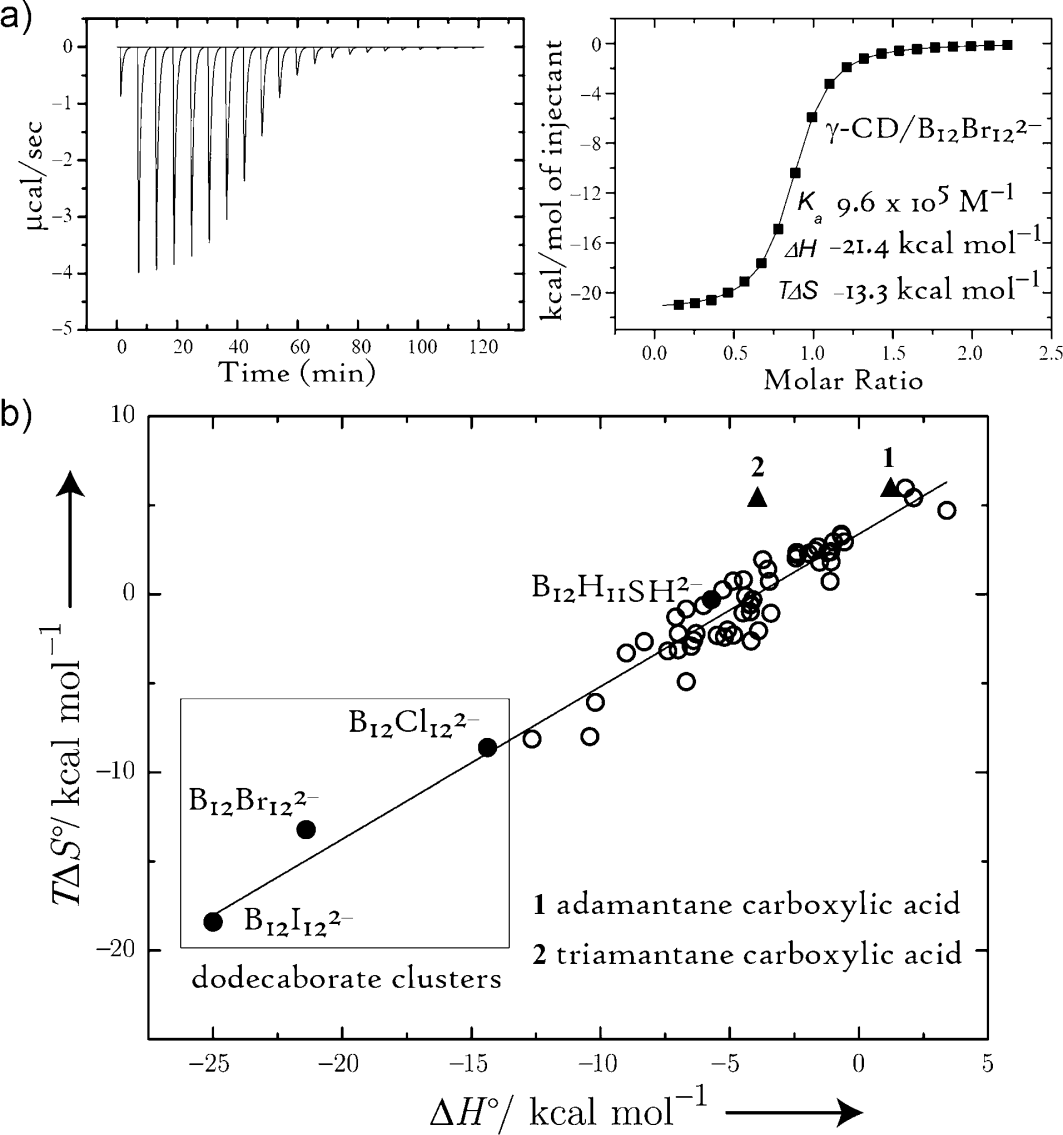
a) Microcalorimetric titration results in neat water: Raw ITC data for sequential injections of 1.0 mm Na_2_B_12_Br_12_ into a γ-CD solution (0.07 mm, left) and apparent reaction heats obtained from the integration of the calorimetric traces (right). b) Enthalpy–entropy compensation plot for γ-CD complexes with dodecaborate anions and previously reported γ-CD complexes with diverse organic guest; data taken from Refs. [2c,^7^].

Table 1 contains a remarkable set of data for this native CD and introduces a new and orthogonal host–guest anchor motif (γ-CD⋅B_12_X_12_^2−^). Until now, even association constants on the order of 10^3^ L mol^−1^ have been fairly difficult to achieve for γ-CD,[2b,[c,^12^] because its large cavity lacks the high-energy water content which assists the binding to smaller cavities.[1c The values observed for the halogenated dianions and γ-CD even rival and exceed the values found for β-CD, the putative highest-affinity host among native CD homologues.[2b For example, the binding constants for the highly hydrophobic adamantyl or ferrocenyl residues (carboxylate or ammonium), which present well-known gold standards in the CD field, reach only ca. 3×10^4^ L mol^−1^ for β-CD,[2b,[c,^7^] a value approached or surpassed across the entire dodecaborate series with γ-CD. Undoubtedly, the spherical shape complementarity[13] of the purely inorganic guests and their high polarizabilities (see the Supporting Information), especially the halogenated ones, contribute to these high affinities through an optimization of dispersion interactions.[14] However, as can be seen from Figure 3 b, the globular clusters vary tremendously in size (more than a factor of three) and also in their electrostatic potential, yet their binding constants with γ-CD remain rather constant (only two orders of magnitude variation). It was exactly this relatively low “selectivity” which pointed to an additional (peculiar but generic) driving force for complexation.

Dodecaborate salts are not only highly water-soluble and display negative log(*P*_OW_) values,[15] but the dianions have very negative free energies and enthalpies of hydration (ca. −140 kcal mol^−1^).[16] They are evidently hydrophilic guests[17] such that a hydrophobic effect cannot account for their high affinities. In searching for alternative explanations, we recalled the nature of these clusters as (even if unconventional) anions and inspected precedents for anion binding to CDs.[18] Indeed, the binding of iodide to α-CD was already reported 50 years back[18a and sizable affinities of perchlorate (up to 66 L mol^−1^) were measured later.[18d Detailed studies ruled out a hydrophobic effect as cause of the inorganic anion binding,[18b but showed that the affinities paralleled their position in the Hofmeister series:[18c chaotropic anions (water structure breakers, such as ClO_4_^−^) showed higher affinities than kosmotropic anions (water structure makers, such as HPO_4_^2−^). We therefore followed the idea whether a “chaotropic effect” could be responsible for the high affinities of the dodecaborates.

We conducted classical salting-in experiments to assess the chaotropic nature of the clusters (see the Supporting Information), which had not been scrutinized before. Indeed, they cause a large increase in the solubilty of adenine and riboflavin, two established standards.[19] Moreover, the solubilizing power of dodecaborates exceeds even those of SCN^−^ and PF_6_^−^, two prototypal chaotropic anions. Based on their salting-in propensity, dodecaborates can be classified as “superchaotropic” anions, that is, they reach beyond the traditional Hofmeister scale; they are also the first salting-in agents bearing two negative charges.[20] This finding has important implications for borate cluster chemistry as a whole, which will be subject to follow-up work. We mention here only that there exist numerous indications on their unusual water solvation,[15] strong interactions with lipid membranes or proteins,[21] as well as unusual affinities, especially of the halogenated dodecaborates, to carbohydrate chromatography matrices,[22] all of which now appear in the new light of their superchaotropic character. The superchaotropic nature of the B_12_X_12_^2−^ clusters was independently confirmed by applying the semiempirical ionic solvation model developed by Marcus (see the Supporting Information).[23]

Based on these new lines of evidence, we conclude that the complexation of dodecaborate dianions is driven by a chaotropic effect.[24] Although the chaotropic effect has in common with the hydrophobic effect that the involved guests are weakly hydrated (compared to kosmotropes or hydrogen-bonding solutes), they are conceptually distinct in that chaotropic anions interfere qualitatively differently with the water structure than hydrophobic species do.[25] The contrasting hydration behavior is borne out by the diametrically opposed thermodynamic fingerprints of the borate clusters versus hydrophobic residues (triangular data points in Figure 4 b).

In mechanistic detail, chaotropic ions decrease the water structure in their surrounding, with two immediately relevant consequences: The water structural entropies for ionic hydration are positive, and there is an effective loss of hydrogen bonds around the anion. Both effects, which can also be modelled according to Marcus (see the Supporting Information), should be particularly pronounced for the dodecaborate cluster dianions. Upon relocation of chaotropic anions from the aqueous bulk into nonpolar binding pockets a significant recovery of the structure of the water network must take place, which should contribute a pronounced loss in water structural entropy and a gain in enthalpy as a consequence of the restoration of hydrogen bonds.

The observed negative complexation entropies for the dodecaborate clusters (Table 1) are indeed on the order of what is expected from the water structural entropies estimated for chaotropic anions (see the Supporting Information). The correlated large negative complexation enthalpies require energetic stabilizations, which are sufficiently large to overcome the concomitant decrease in ion-dipole interactions. They can be accounted for in terms of 1) the reformation of the broken hydrogen bonds in the aqueous bulk upon binding of the chaotropic anions and 2) increased dispersion interactions of the guests with the host than with water. That the recovery of hydrogen bonds presents an important supramolecular driving force is known from the release of high-energy water from small macroyclic cavities,[1c while the importance of dispersion interactions can be deduced from the very high polarizabilities calculated for the borate clusters (see the Supporting Information). Since both enthalpic effects increase indirectly[26] or directly[1c,^27^] with the polarizability of the anions, and because the chaotropic nature of anions increases with their size and polarizability, the described chaotropic effect includes implicitly contributions from dispersion.[28]

It transpires that the chaotropic effect pin-pointed here describes a generic driving force for the encapsulation of chaotropic anions into suitable sized organic cavities in aqueous solution, with the propensity: B_12_X_12_^2−^(new)≫PF_6_^−^>ClO_4_^−^>SCN^−^, I^−^>Br^−^≫ kosmotropes. Chaotrope encapsulation results in an effective water structure recovery and is enthalpically driven, with an invariably negative entropic component as fingerprint. The chaotropic effect accounts for previous (for CDs)[29] and very recent (for CDs and other macrocycles)[30] observations on the high-affinity binding of such anions, and it merges independent, consistent observations for the same anions to be driven to interfaces,[20],26b,^31^] to penetrate into lipid bilayers,[20],^32^] and to bind in protein binding pockets.[33]
