# Supplementary material for: Water Structure Recovery in Chaotropic Anion Recognition: High-Affinity Binding of Dodecaborate Clusters to γ-Cyclodextrin
Source: Angew Chem Int Ed Engl. 2015 May 7;54(23):6852–6. doi: 10.1002/anie.201412485 (PMC4510780; doi:10.1002/anie.201412485)
Supplement: Supplementary file 1 [file anie0054-6852-sd1.pdf]

Angewandte Chemie
Eine Zeitschrift der Gesellschaft Deutscher Chemiker

Supporting Information

German Edition: DOI:

Water Structure Recovery in Chaotropic Anion Recognition: High-Affinity Binding of Dodecaborate Clusters to γ-Cyclodextrin**

Khaleel I. Assaf, Merve S. Ural, Fangfang Pan, Tony Georgiev, Svetlana Simova, Kari Rissanen, Detlef Gabel,* and Werner M. Nau**

anie_201412485_sm_miscellaneous_information.pdf

Table of Contents

1. Materials and methods 1
2. Binding affinities of other studied complexes 2
3. 2D ROESY ^{1}H NMR spectra 3
4. X-ray crystallography 6
5. Quantum-chemical calculations 10
6. Isothermal titration calorimetry (ITC) 11
7. ^{1}H NMR spectra 12
8. Job's plots 17
9. Solubilization experiments 18
10. Ion properties 20
11. References 22

1. Materials and methods

The boron clusters were synthesized according to published procedures.[1-3] Cyclodextrins were purchased from Sigma-Aldrich and used without further purification. Octakis(6-Amino-6-Deoxy)-γ-Cyclodextrin was obtained from CycloLab. NMR spectra were recorded with a JEOL ECX 400 MHz and a Brucker A VII 600MHz NMR spectrometer in D_2O (99.9 %, from Aldrich). Isothermal titration calorimetry experiments were carried out on a VP-ITC from Microcal, Inc., at 25 °C. The binding equilibria were studied using a cellular γ-CD concentration of 0.07 mM (after correction for 25% water content in the commercial γ-CD sample), to which a 10-30 times more concentrated guest solution was titrated. Typically, 20–30 consecutive injections of 10 μL were used. All solutions were degassed prior to titration. Heats of dilution were determined by titration of the dodecaborate cluster solution into water. The first data point was removed from the data set prior to curve fitting with Origin 7.0 software according to a one-set-of-sites model. The knowledge of the complex stability constant (K_a) and molar reaction enthalpy ($\Delta H°$) enabled the calculation of the standard free energy ($\Delta G°$) and entropy changes ($\Delta S°$) according to $\Delta G° = -RT \ln K_a = \Delta H° - T\Delta S°$.

2. Binding affinities of other studied complexes

The smaller α-CD and β-CD homologues showed smaller changes in the ^{1}H NMR spectra, e.g., 0.01, 0.05, and 0.09 ppm down-field shift of the H-3 proton for α-, β-, and γ-CD as host and $B_{12}H_{11}SH^{2-}$ as guest (see Section 7); the association constants resulting from the host-guest titrations followed the same order (compare Table S1 with Table 1 in main text). The affinity trend, namely γ-CD >> β-CD > α-CD can be readily understood in terms of the known size selectivity for inclusion complexes, i.e., the size of the clusters is too large to be effectively encapsulated into the smaller CD cavities.

Table S1. Binding constants of other studied complexes, measured by ^{1}H NMR.

Host	Guest[a]	K_a/ (10^3 M^{-1})
α-CD	$B_{12}H_{12}^{2-}$	0.1
β-CD	$B_{12}H_{12}^{2-}$	0.2
α-CD	$B_{12}H_{11}SH^{2-}$	0.1
β-CD	$B_{12}H_{11}SH^{2-}$	1.0
Me-β-CD	$B_{12}H_{11}SH^{2-}$	n.d.
Hydroxylethyl-β-CD	$B_{12}H_{11}SH^{2-}$	n.d.
β-CD	$B_{12}Br_{12}^{2-}$	0.5
NH_3^+-γ-CD	$B_{12}H_{11}SH^{2-}$	> 20
NH_3^+-γ-CD	$B_{12}Br_{12}^{2-}$	> 60
β-CD	$B_{12}I_{12}^{2-}$	0.4
γ-CD	$B_{12}H_{11}OR^{2-}$ [b]	2.0

[a] As sodium salts, unless stated differently.
[b] R = $(CH_2)_4C(NH_2)CO_2H$, as cesium salt.

3. 2D ROESY ^{1}H NMR spectra

^{1}H NMR interactions between the B–H protons and the CD protons are less informative, due to the extreme broadening of the B–H resonance (from −0.5 to +2.0 ppm, Figures S1-4). Most functional groups (OH, SH, NH2) could not be used either to pinpoint their positioning in the complexes. However, for $B_{12}H_{11}NR_3^-$ (with R = Me, Et, *n*Pr, *n*Bu), we observed 2D-ROESY cross-peaks between the aliphatic protons and the H-3 proton of γ-CD, that is, the functional group is positioned near the wider rim (Figure S1-4).

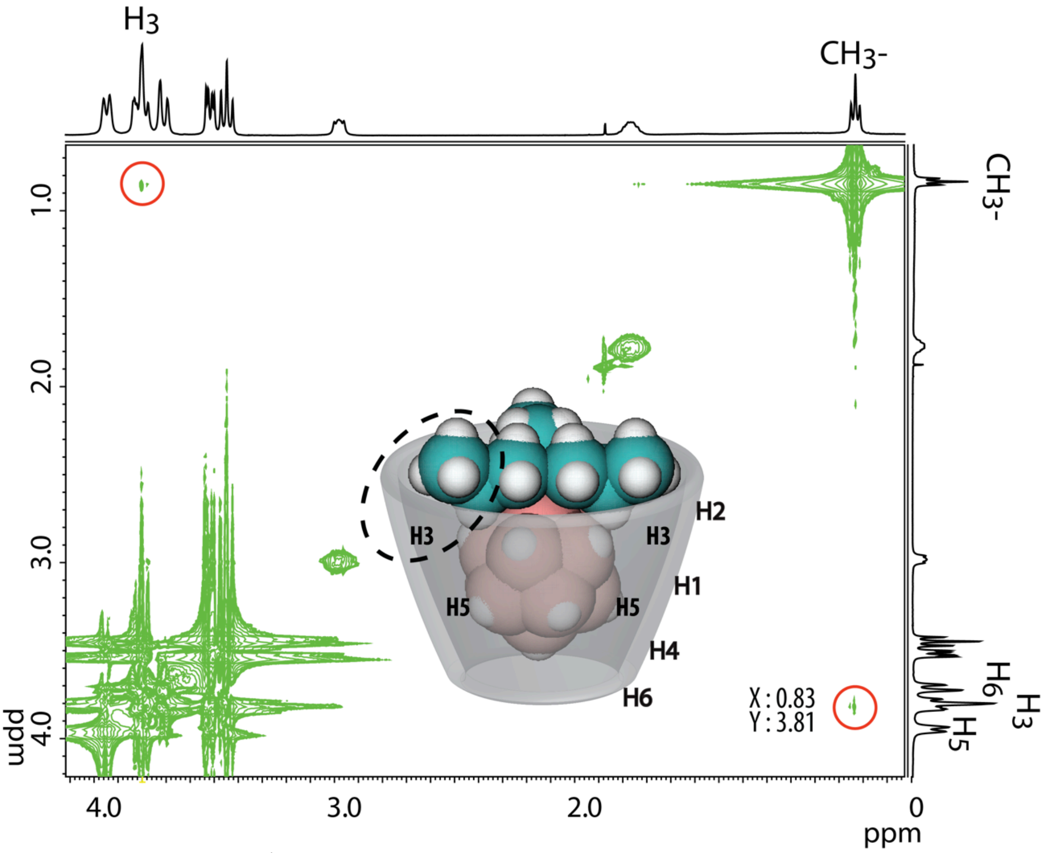

Figure S1. 2D ROESY ^{1}H NMR spectral section for a 1:1 mixture of $B_{12}H_{11}N(nPr)_3^-$ (as potassium salt) with γ-CD in D_2O. Inset: Structural assignment of the inclusion complex; circles indicate cross-peaks.

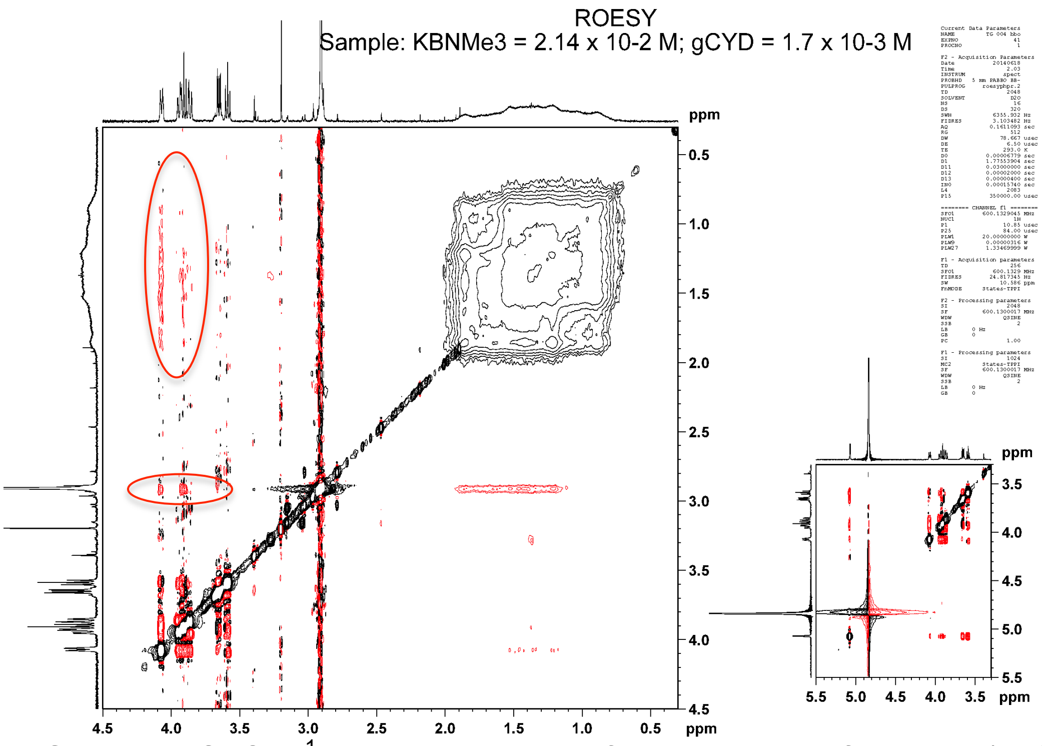

Figure S2. 2D ROESY ^{1}H NMR spectrum for a mixture of $B_{12}H_{11}N(Me)_3^{-}$ (as potassium salt) with γ-CD in D_2O; ellipses indicate cross-peaks.

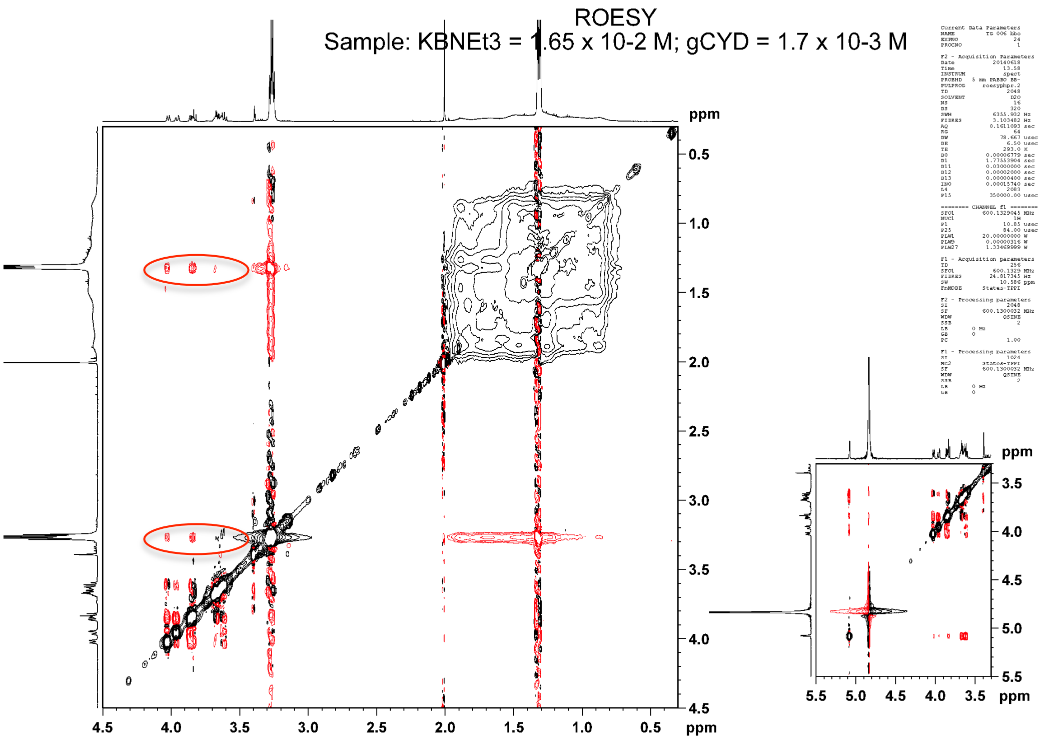

Figure S3. 2D ROESY ^{1}H NMR spectrum for a mixture of $B_{12}H_{11}N(Et)_3^{-}$ (as potassium salt) with γ-CD in D_2O; ellipses indicate cross-peaks.

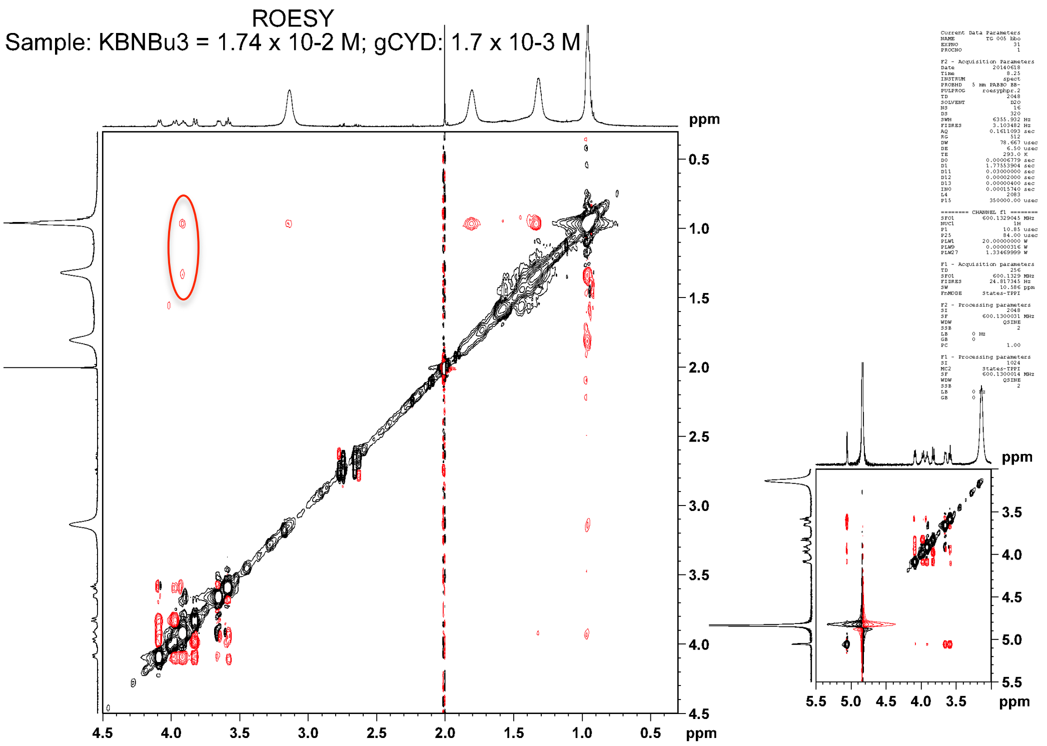

Figure S4. 2D ROESY ^{1}H NMR spectrum for a mixture of $B_{12}H_{11}N(nBu)_3^{-}$ (as potassium salt) with γ-CD in D_2O; ellipses indicate cross-peaks.

4. X-ray crystallography

Single crystals of the γ-CD complex with $B_{12}Br_{12}^{2-}$ were obtained by slow diffusion of methanol (over three days) into a satutarted aqueous solutions of γ-CD and $Na_2B_{12}Br_{12}$. Single-crystal X-ray diffraction analysis was performed at 123.0(1) K on an Agilent Super-Nova diffractometer using mirror-monochromatized Cu-*K*α (λ = 1.54184 Å) radiation. The *CrysAlisPro*[4] program was used for data collection and processing. The intensities were corrected for absorption by using the analytical face index absorption correction method.[5] The structure was solved with *XM*[6] and refined by full-matrix least-squares methods by means of the *OLEX2*[7] software, which utilizes the *SHELXL-2013* module.[6] The dianionic $B_{12}Br_{12}^{2-}$ cluster was located on a 4-fold symmetry axis, severely disordered, and was modelled by assuming a three-position disorder. The minor disordered components of the boron atoms in the cluster were isotropically refined with fixed thermal parameters. All the other non-hydrogen atoms were refined with anisotropic displacement parameters. Hydrogen atoms in the γ-CD molecules were introduced in proper positions with isotopic thermal vibration by using the 'riding model', while those for the solvent water molecules were constrained and fixed in positions with suitable hydrogen bonds. Some of the solvent water molecule H-atoms were not located due to disorder. Additionally, SQUEEZE[8] was used to exclude electrons in the voids of the structure, because the residual electron densities were too weak to make any chemically reasonable model. Application of the SQUEEZE method resulted in a crystallographically unreasonable lattice with very large voids (see Figure S5 (A)). The gap between the resolved cyclodextrin moieties was so big that it could only be explained by a severly disordered fourth cyclodextrin molecule. To probe if this is indeed the case, a model was developed, in which electron densities higher than 0.6 e/Å^3 in the difference Fourier Map were assigned to either carbon or oxygen atoms. The isotropic thermal parameters of these 'dummy" C- and O-atoms were constrained to be 0.15 but the occupancies of each dummy atom were allowed to refine freely. By comparing these two models, combined with the PLATON[8] VOIDS estimated electron count in the voids (1460 e/ cell) and the solvents used for crystallization, it can be concluded that the large voids in the SQUEEZED structure are actually occupied by two additional, highly disordered γ-CDs and 36 water molecules in each unit cell. This resulted in the overall composition of the crystal to be 2{Na_2[γ-CD…$B_{12}Br_{12}$…γ-CD]} × 3 γ-CD × $n$$H_2O$, and specifically modelled as 3.5($C_{48}H_{80}O_{40}$)●$B_{12}Br_{12}$●2Na●60(H_2O). Table S2 reports the differences between the SQUEEZED and the "dummy atom" refinements.

The graphical representation (Figure 3a in the main text) of the $B_{12}Br_{12}^{2-}$ cluster was done by replacing the severely disordered cluster from the SQUEEZED data by the same cluster from the X-ray structure coordinates of bis(benzyltriethylammmonium) dodecabromo-closo-dodecaborate[9] (CCDC code GAPJIR) and placing it exactly at the same place inside the dimeric CD motif as it occurs in the SQUEEZED data to produce a non-disordered image of the [γ-CD···$B_{12}Br_{12}$···γ-CD]CD inclusion complex motif.

Table S2. Crystallographic data and data refinement results.

γ-CD_$B_{12}Br_{12}^{2-}$		
Data<br>CCDC deposit number	- Squeezed -<br>1022517	- Unsqueezed with 'dummy atom' assignments for the electron densities in voids -
Chemical formula	$3(C_{48}H_{80}O_{40})\bullet B_{12}Br_{12}\bullet 2Na\bullet 35(H_2O)$	$3.5(C_{48}H_{80}O_{40})\bullet B_{12}Br_{12}\bullet 2Na\bullet 60(H_2O)$
M_r	5656.46	6755.40
Crystal system, space group	Tetragonal, *I*422	
Temperature/K	123.0(1)	
a, *c* (/Å)	23.7594(5), 56.732(2)	
V/Å^3	32026(2)	
Z	4	
Radiation type	CuK_α	
μ/mm^{-1}	2.56	
Crystal size/mm	0.10 × 0.08 × 0.03	
Data collection		
Diffractometer	SuperNova, Dual, Cu at zero, Atlas detector	
T_{min}, T_{max}	0.963, 0.984	
No. of measured, independent and observed [$I > 2\sigma(I)$] reflections	35541, 14115, 6469	35541, 14115, 6558
R_{int}	0.074	0.077
$(\sin \theta/\lambda)_{max}$ (/Å^{-1})	0.596	0.596
Refinement		
$R[F^2 > 2\sigma(F^2)]$, $wR(F^2)$, S	0.118, 0.343, 0.99	0.114, 0.338, 0.98
No. of parameters	907	1135
No. of restraints	132	121
$\Delta\rho_{max}$, $\Delta\rho_{min}$ (/e Å^{-3})	0.79, −0.83	0.66, −0.77
Absolute structure[10]	Flack x determined using 2057 quotients [(I+)−(I−)]/[(I+)+(I−)]	Flack x determined using 2088 quotients [(I+)−(I−)]/[(I+)+(I−)]
Flack parameter	0.13(2)	0.06(2)
Computer programs	*CrysAlis PRO,*[4] XM,[6] *SHELXL,*[6] Olex2[7]	

Crystal packing. The γ-CD macrocycles pack in the unit cell in parallel stacks forming a formal tubular crystal lattice (Figure S6) along the *c* axis. In each stack, the γ-CDs are linked either by intermolecular hydrogen bonds or by Na···O bonds to the adjacent stacks. The detailed analysis of the crystal lattice reveals two γ-CD motifs (Figure S6). The first is a capsular head-to-head (upper rim-to-upper rim) dimer encapsulating the highly disordered $B_{12}Br_{12}^{2-}$ cluster. Within each stack, these cluster-encapsulating CD dimers are separated from each other by three solvent-filled CDs, of which the central CD is severely disordered. The interstices between the stacks are filled by solvent water molecules, many of which are disordered. In this way, in the crystal lattice, the CDs within the stacks are connected through CD-to-CD or H_2O-mediated hydrogen bonds, resulting in a formal 7:2 ratio of the closed packed CDs and the entrapped dodecaborate cluster, supplemented with a large number of water molecules.

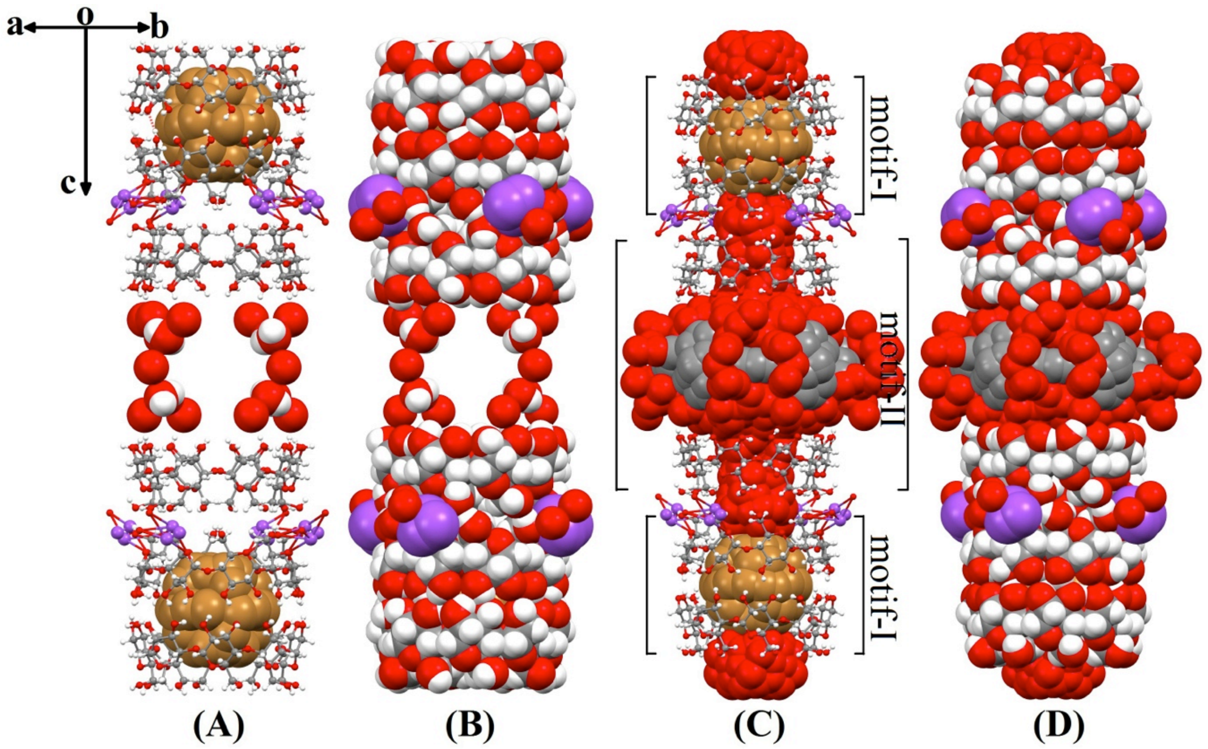

Figure S5. Combination of the ball-and-stick model and the space-filling model to show that the voids in the tube are presumably occupied by heavily disordered γ-CD and solvent water molecules (C = gray, O = red, H = white, Na = purple, Br = brown, B = pink). (A) and (B) refer to the lattice with SQUEEZED data, and (C) and (D) refer to the “dummy” atom model with the original data.

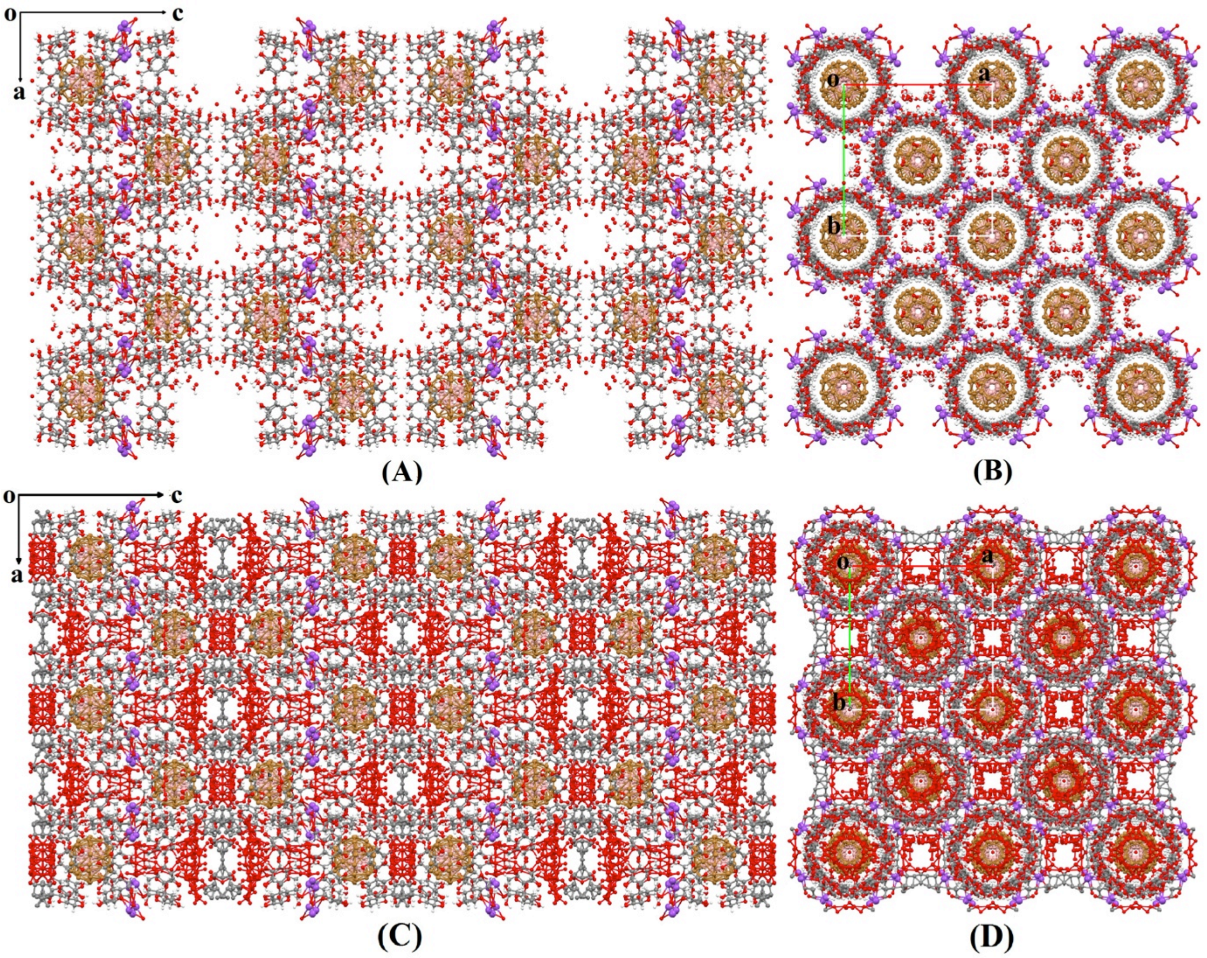

Figure S6. Packing of the γ-CD molecules (C = gray, O = red, H = white), sodium cations (purple), $B_{12}Br_{12}^{2-}$ clusters (Br = brown, B = pink), and water molecules (O = red). The lattice from the SQUEEZED data viewed in the *ac* plane (A) and *ab* plane (B). The lattice from the "dummy" atom data viewed in the *ac* plane (C) and *ab* plane (D).

5. Quantum-chemical calculations

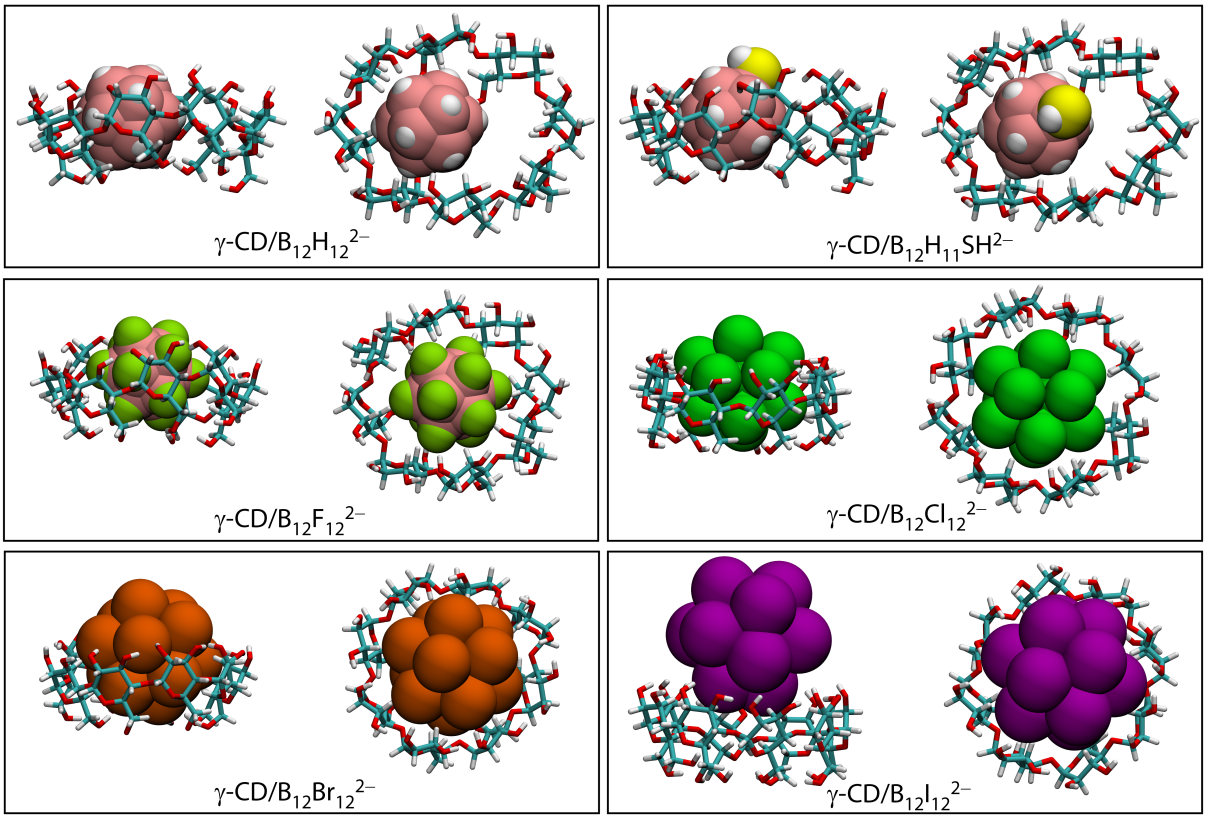

Figure S7. PM3-optimized geometries of different dodecaborate cluster/γ-CD complexes illustrating relative size relationships and trends.

The polarizabilities of selected dodecaborate clusters and some reference compounds were calculated by means of DFT calculations (Table S3). The high polarizabilities of the clusters are attributed to their di-anionic nature, their quasi-3D-aromatic character, and the displacement of the negative partial charges towards the cluster surface as a result of electronegativity differences (even in B–H bonds).

Table S3. Calculated polarizabilities (in Å^3) of dodecaborate anions, calculated in the gas phase by using the B3LYP/aug-cc-pvdz method.

	Cl^-	Br^-	I^-	adamantane	$B_{12}H_{12}^{2-}$	$B_{12}Cl_{12}^{2-}$	$B_{12}Br_{12}^{2-}$	$B_{12}I_{12}^{2-}$
Calc.	3.5	4.8	7.9	16.2 [15.5][b]	25.7 [25.6][b,d]	46.7	58.2	84.1[e]
Exp.	3.25[a]	4.53[a]		15.9[c]				

[a] From ref. 11. [b] Calculated at the B3LYP/6-31+G* level of theory. [c] Experimental value from ref. 12. [d] Calculated value by using DFT calculations in a dielectric-continuum model for acetonitrile from ref. 13: 36.5 Å^3. [e] Calculated by using Dunning's correlation-consistent polarized valence double-zeta basis set augmented with diffuse functions and employing pseudopotentials to describe the inner core orbitals of the iodine atoms.

6. Isothermal titration calorimetry (ITC)

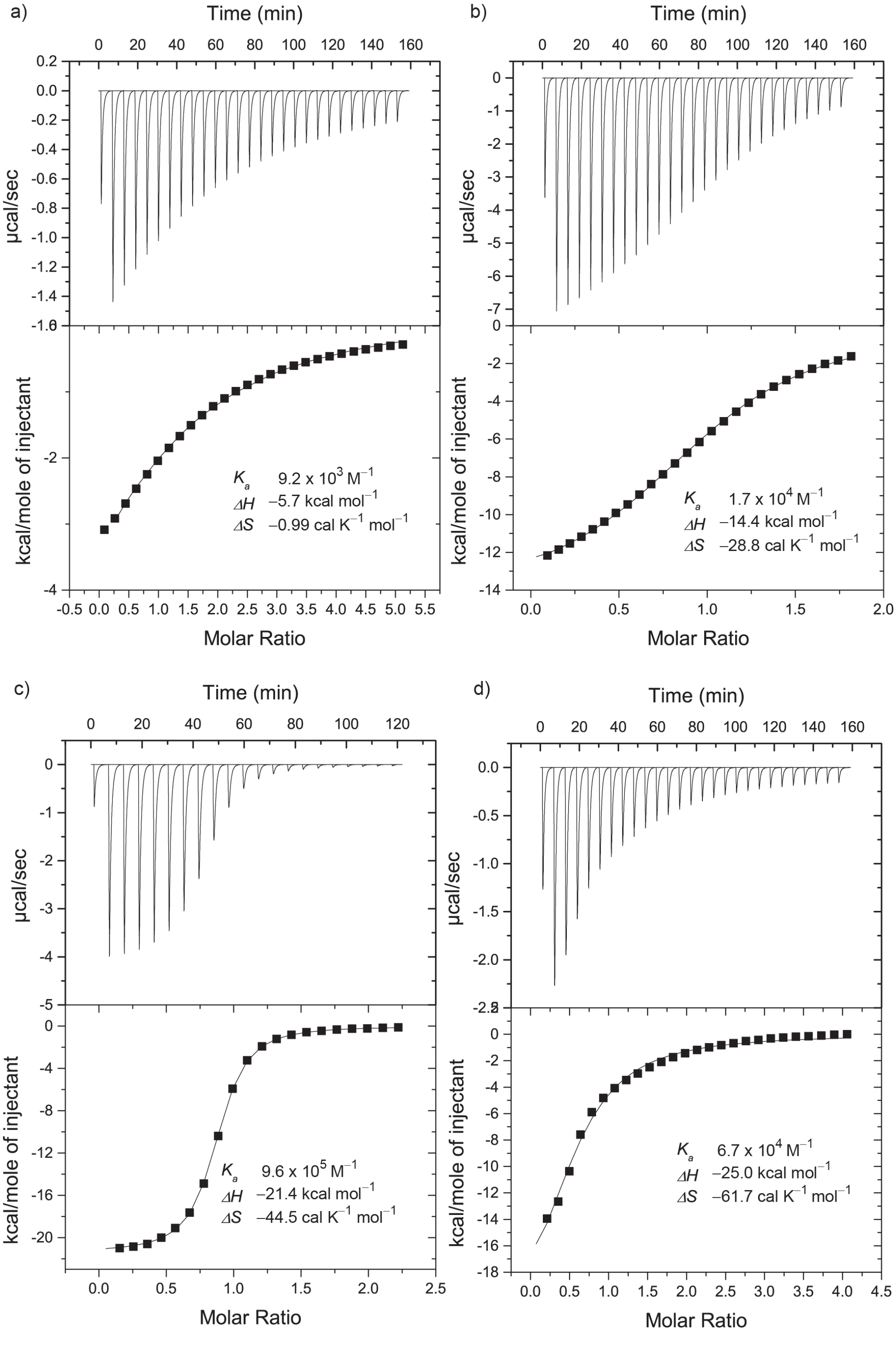

Figure S8. Microcalorimetric titration of γ-CD with a) $Na_2B_{12}H_{11}SH$, b) $Na_2B_{12}Cl_{12}$, c) $Na_2B_{12}Br_{12}$, and d) $Na_2B_{12}I_{12}$. Top) Raw ITC data for sequential twenty-seven injections of guest solution into the host solution. Bottom) Apparent reaction heats obtained from the integration of calorimetric traces. Guest/host concentrations in mM: a) 3.14/0.070, b) 3.10/0.35, c) 1.00/0.070, and d) 1.00/0.070. The stoichiometric *n* values were throughout within the range of 1.0±0.2.

7. ^{1}H NMR spectra

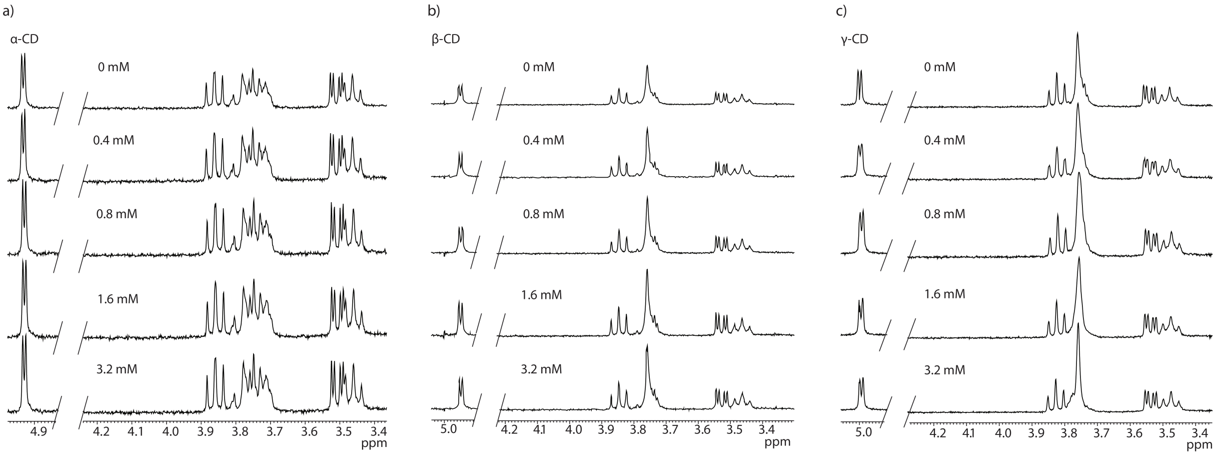

Figure S9. ^{1}H NMR spectra of 0.175 mM of a) α-CD b) β-CD and c) γ-CD with different concentrations of $Na_2B_{12}H_{12}$.

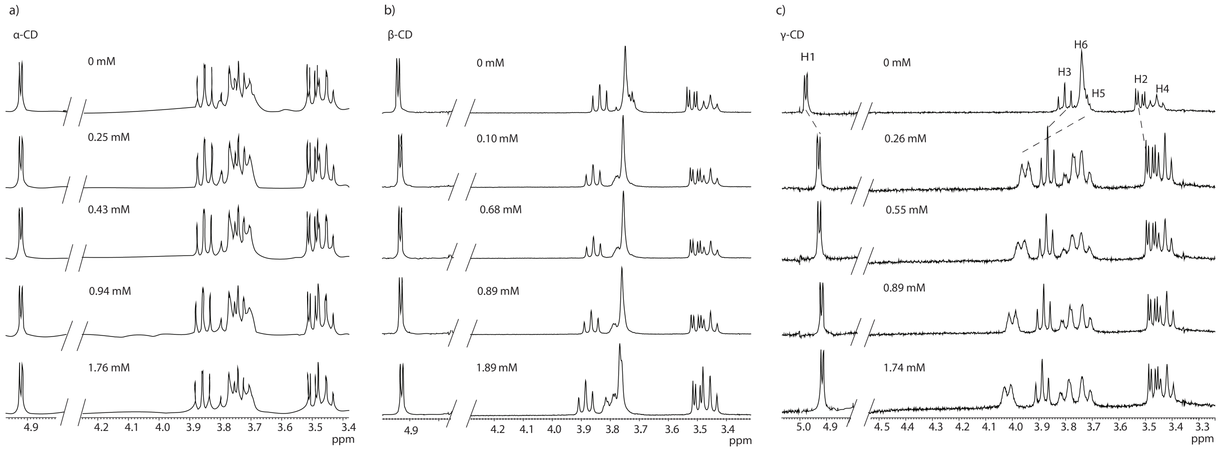

Figure S10. ^{1}H NMR spectra of a) 0.7 mM of α-CD, b) 0.7 mM of β-CD, and c) 0.17 mM of γ-CD with different concentrations of $Na_2B_{12}H_{11}SH$.

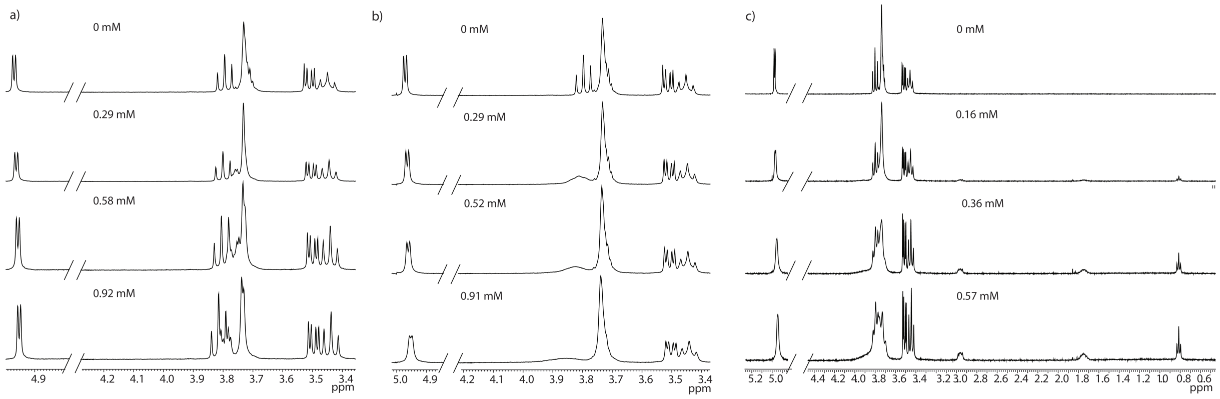

Figure S11. ^{1}H NMR spectra of 0.7 mM γ-CD with different concentrations of a) $Na_2B_{12}H_{11}OH$, b) $NaB_{12}H_{11}NH_3$, and c) $KB_{12}H_{11}N(CH_2CH_2CH_3)_3$.

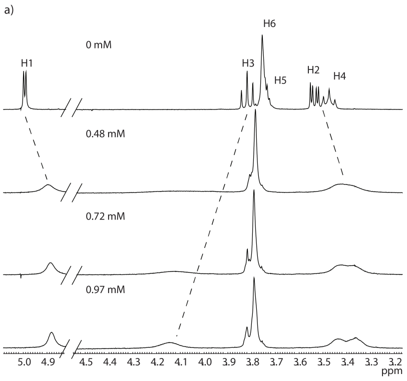

Figure S12. ^{1}H NMR spectra of 0.7 mM of γ-CD with different concentrations of a) $Na_2B_{12}Cl_{12}$, b) $Na_2B_{12}Br_{12}$, and c) $Na_2B_{12}I_{12}$.

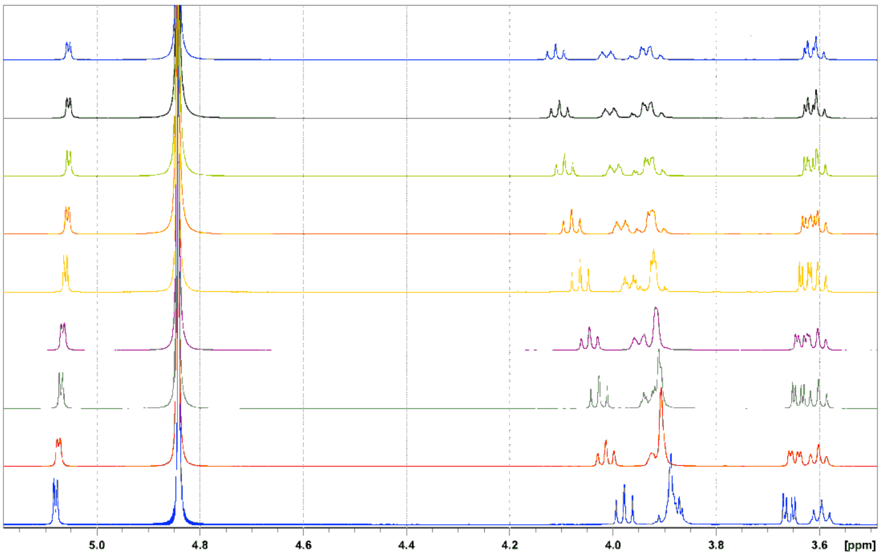

Figure S13. ^{1}H NMR titration of 10 mM γ-CD with different concentrations of $Na_2B_{12}H_{12}$, from bottom (0 mM) to top (46 mM).

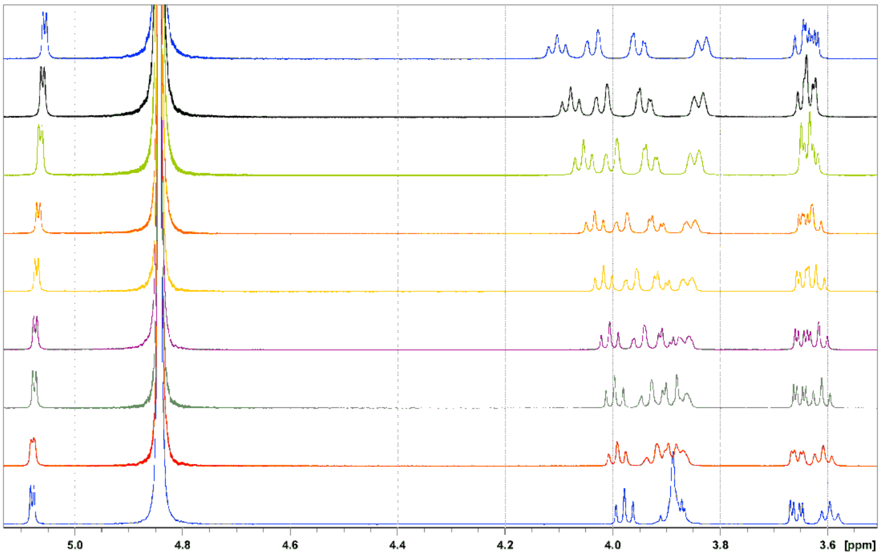

Figure S14. ^{1}H NMR titration of 2 mM β-CD with different concentrations of $Na_2B_{12}I_{12}$; from bottom (0 mM) to top (9 mM).

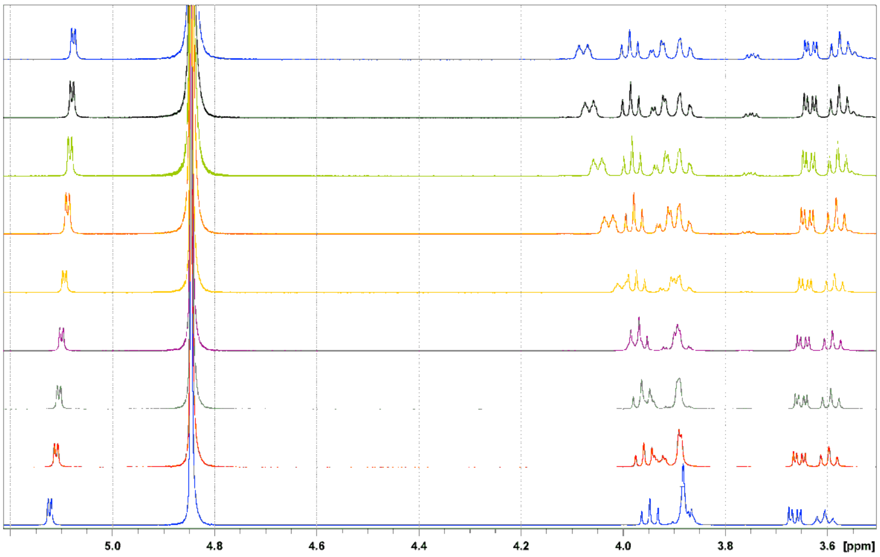

Figure S15. ^{1}H NMR titration of 1 mM γ-CD with different concentrations of $Cs_2B_{12}H_{11}O(CH_2)_4C(NH_2)CO_2H$, from bottom (0 mM) to top (5 mM).

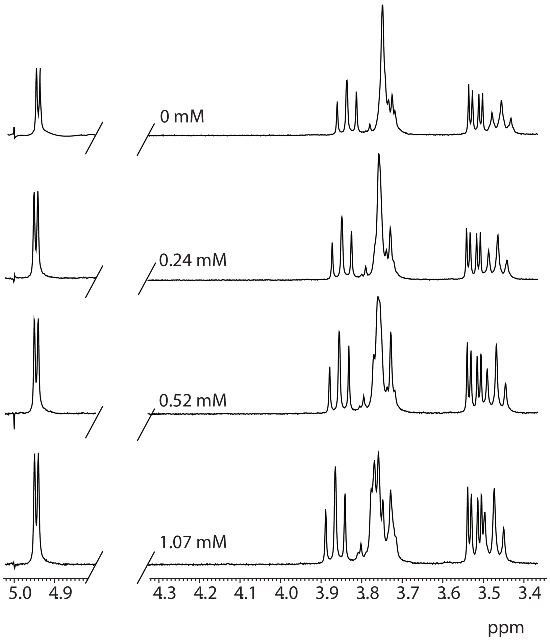

Figure S16. ^{1}H NMR spectra of 0.7 mM β-CD with different concentrations of $Na_2B_{12}Br_{12}$.

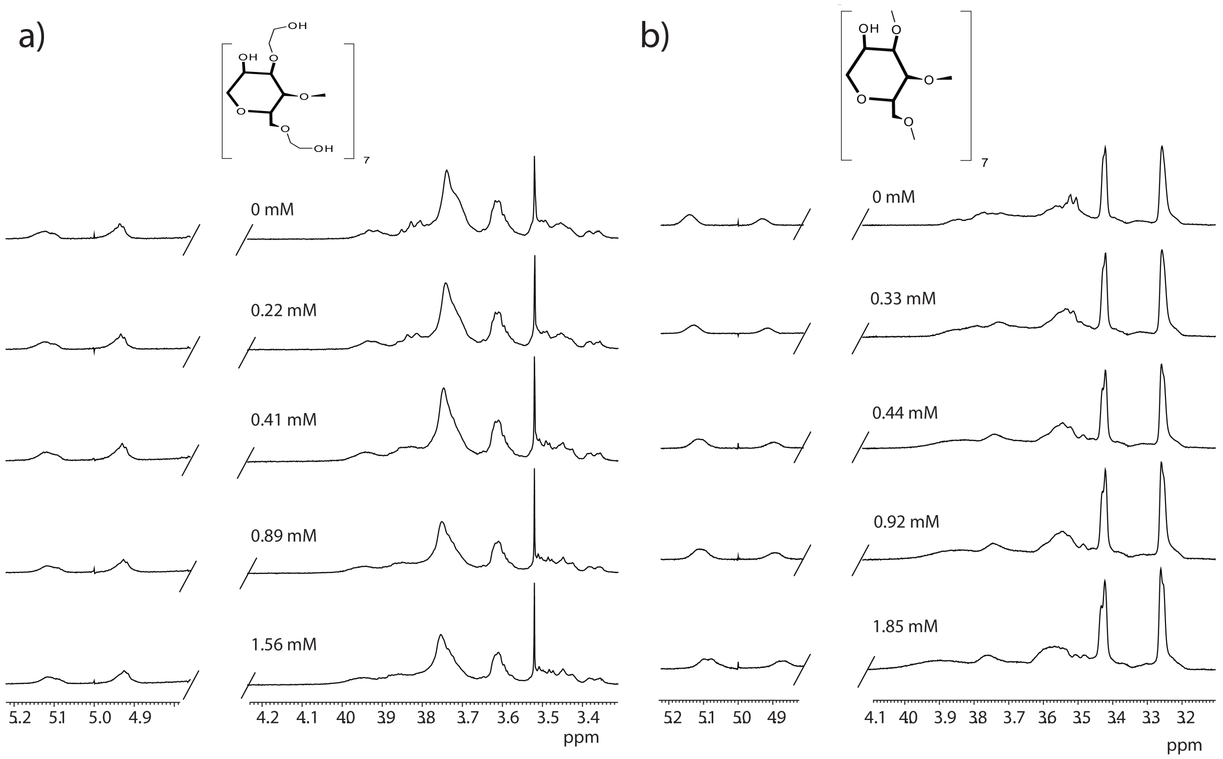

Figure S17. ^{1}H NMR spectra of a) 0.7 mM methyl-β-CD with different concentrations of $Na_2B_{12}H_{11}SH$ and b) 0.7 mM hydroxylethyl-β-CD with different concentrations of $Na_2B_{12}H_{11}SH$.

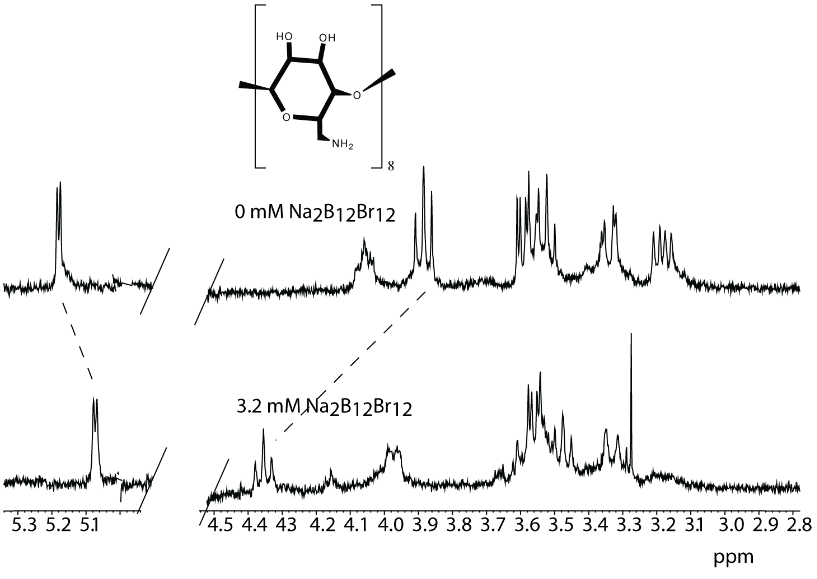

Figure S18. ^{1}H NMR spectra of 0.175 mM NH_3^+γ-CD with $Na_2B_{12}Br_{12}$.

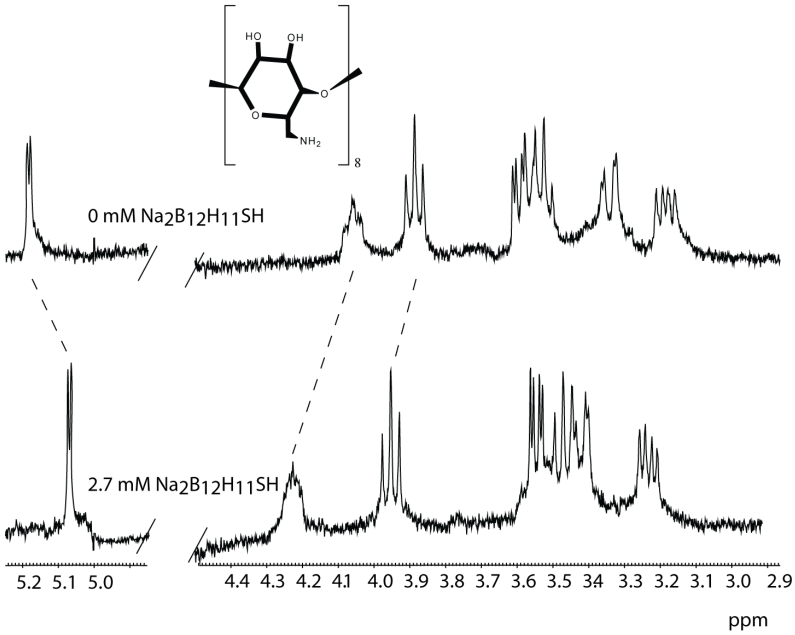

Figure S19. ^{1}H NMR spectra of 0.175 mM NH_3^+γ-CD with $Na_2B_{12}H_{11}SH$.

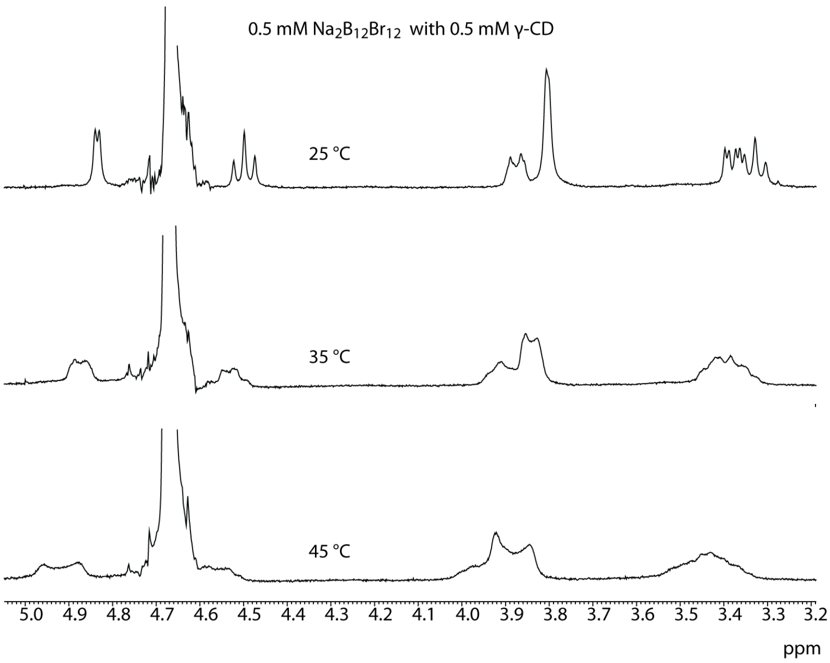

Figure S20. ^{1}H NMR spectra of 0.5 mM γ-CD with 0.5 mM of $Na_2B_{12}Br_{12}$ at different temperatures.

8. Job's plots

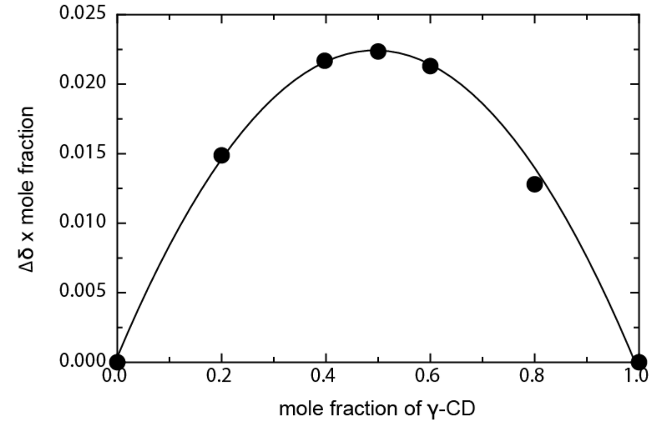

Figure S21. Job's plot for γ-CD with $Na_2B_{12}H_{11}SH$.

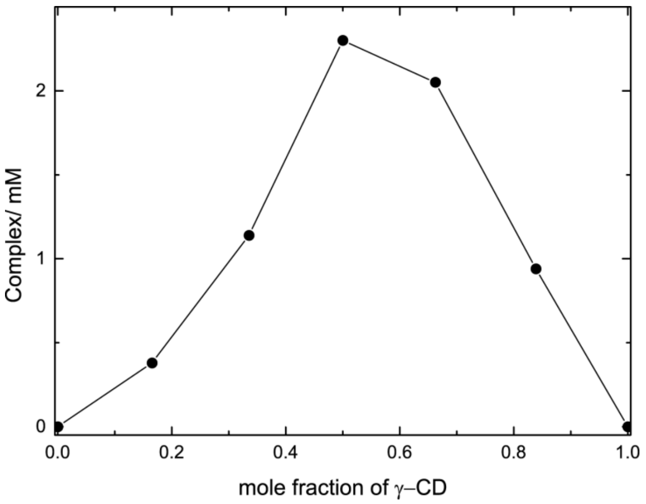

Figure S22. Job's plot for γ-cyclodextrin with $Na_2B_{12}Br_{12}$. The complex exhibits slow exchange, such that relative integrals (converted to concentrations of complex) instead of averaged chemical shifts (as in Figure S21 and S23) were plotted; the spiked appearance is likely to derive from this different analysis method.

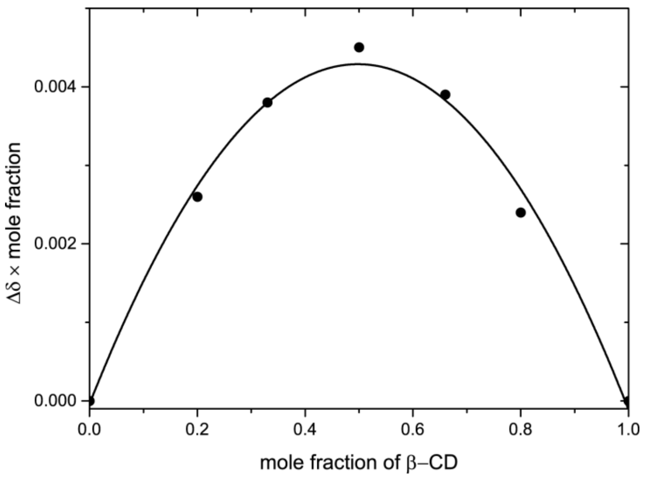

Figure S23. Job's plot for β-cyclodextrin with $Na_2B_{12}I_{12}$.

9. Solubilization experiments

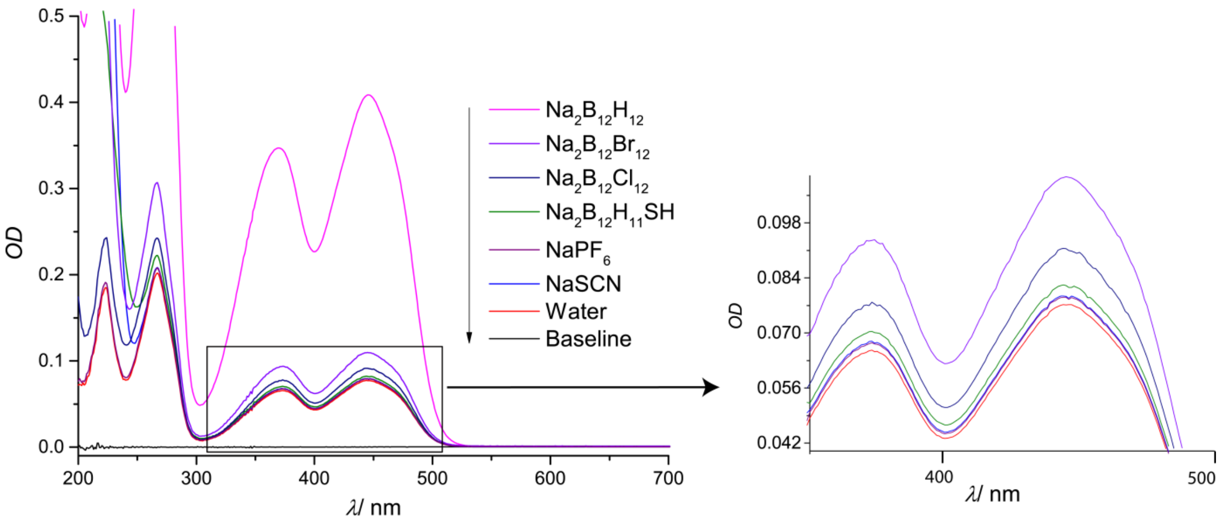

Figure S24. UV-Visible spectra of riboflavin in the presence of 10 mM salts. 1 mg riboflavin was added to solutions of 10 mM salts; after shaking for 10 minutes, the solutions were filtered and measured at 10-fold dilution. The parent cluster ($Na_2B_{12}H_{12}$) caused NMR shifts of riboflavin, indicative of direct interactions; this cluster was therefore neglected in the analysis of the trend (see inset on right).

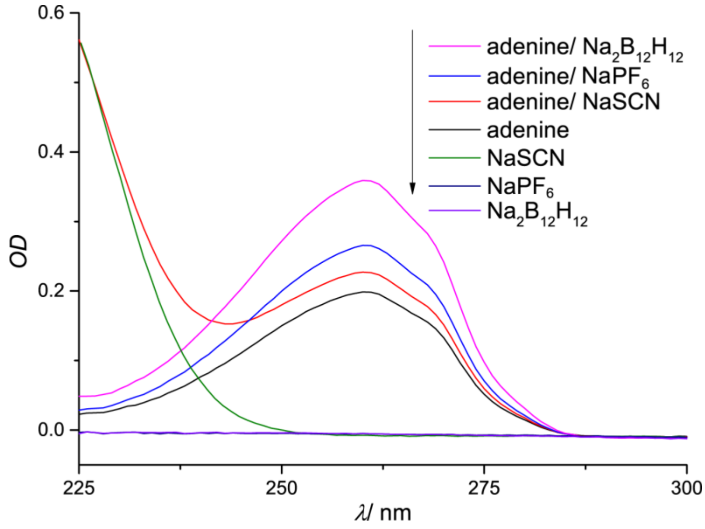

Figure S25. UV-Visible spectra of adenine in the presence of 100 mM salts; 1 mg riboflavin was added to solutions of 100 mM salts in 10 mM phosphate buffer (pH 7.2); after shaking for 10 minutes, the solutions were filtered and measured at 400-fold dilution.

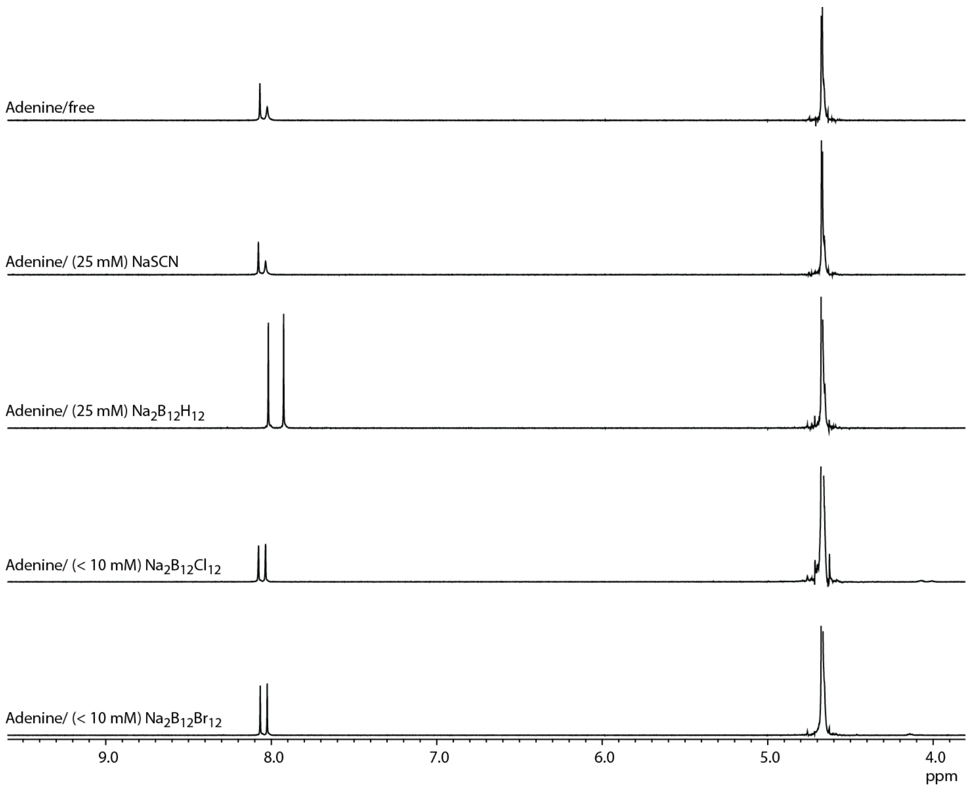

Figure S26. ^{1}H NMR spectra of adenine in the presence of different salts; solid adenine was added to solutions containing the salts. Note the enhanced integrals near 8 ppm as a measure of the relative concentration of dissolved analyte in the presence of the borate clusters. Note also the up-field shift and altered coupling constant for the parent cluster ($Na_2B_{12}H_{12}$), indicative of direct interactions.

10. Ion properties

The chaotropic nature of the $B_{12}X_{12}^{2-}$ clusters was theoretically evaluated according to the semiempirical model of Marcus.[14-17] The model requires as input data (Table S4) the following: a) the ionic radii (r) as tabulated or derived from calculated geometries, b) the width of the hydration shell (Δr) as calculated from eq. 3 in ref. 14, c) the average number of water molecules (n) in the hydration shell as calculated from eq. 2 in ref. 14, and d) the experimental thermochemical properties for the hydration of the ions, in particular their hydration entropies. It should be noted that Δr and n are model-based parameters, and not meant to realistically reproduce the actual hydration pattern.

Table S4. The radius, r, width of hydration shell, Δr, number of water molecules in the hydration shell, n, and hydration entropies, $\Delta_{hydr}S_{exp}$, of different anions

Ion	r/pm [a]	Δr/pm[b]	n[b]	$\Delta_{hydr}S_{exp}$/ (cal K^{-1} mol^{-1}) [c]
F^-	133	78.5	2.7	–32.8
Cl^-	181	42.5	2.0	–17.9
Br^-	196	35.2	1.8	–14.1
I^-	220	26.3	1.6	–8.6
I_3^-	285	13.0	1.3	–17.5
SCN^-	213	28.6	1.7	–15.8
BF_4^-	230	23.5	1.6	–15.7
ClO_4^-	240	20.9	1.5	–13.6
BPh_4^-	421	4.2	0.9	–73.7
PF_6^-	295	11.8	1.2	- - -
$H_2PO_4^-$	238	21.4	1.5	–39.7
NO_3^-	179	43.5	2.0	–18.2
$S_2O_3^{2-}$	250	35.2	2.9	–43.0
SO_4^{2-}	230	43.2	3.1	–59.6[d]
CO_3^{2-}	178	75.4	4.0	–58.6
HPO_4^{2-}	238	39.8	3.0	–65.0
$B_{12}H_{12}^{2-}$	400[e]	9.6	1.8	–17.9[f]
$B_{12}Cl_{12}^{2-}$	525[e]	4.3	1.4	- - -
$B_{12}Br_{12}^{2-}$	560[e]	3.6	1.3	- - -
$B_{12}I_{12}^{2-}$	590[e]	3.1	1.2	- - -
PO_4^{3-}	238	56.0	4.5	–100.7

[a] As tabulated in refs 14 and 15, unless stated differently. [b] Calculated data, this work. [c] Experimental data, as compiled in ref 15. [d] Updated value from Table 2.3 in ref. 17. [e] From calculated structures (see Section 5). [f] From ref. 18.

From the ionic properties in Table S4, the Marcus model allows the calculation of several parameters (Table S5), which have been shown to provide measures of the water-structure breaking (chaotropic) nature of ions, namely *i*) the water-structural entropies for ionic hydration (ΔS_{struct}, calculated from eq. 19 and eq. 20 in ref. 16)[19] and *ii*) the change in the number of hydrogen bonds around the anion (ΔHB, calculated as dimensionless values from the ΔS_{struct} values according to eq. 25 in ref. 16, there labeled as ΔG_{HB}). Marcus has shown that these parameters correlate well with experimental measures of their effects on the water structure, for example with the solvent viscosity *B* coefficients.[14] If the ΔS_{struct} values are positive, the water structure in their surrounding decreases, which can be converted into an effective loss of hydrogen bonds around the anion (negative values of ΔHB). This scale developed by Marcus, while based on extrapolations from experimental data for alkali halides, sets up a convenient "chaotropicity" scale, although the absolute numbers need to be interpreted with caution.

Table S5. Water-structural entropies of different anions (ΔS_{struct})[a] and net effects on the number of surrounding hydrogen bonds (ΔHB)[b]

Anion	$T\Delta S_{struct}$/ (kcal mol^{-1})	ΔHB	Anion	$T\Delta S_{struct}$/ (kcal mol^{-1})	ΔHB
PO_3^{3-}	–9.6	1.20	BF_4^-	6.6	–1.12
HPO_4^{2-}	–4.5	0.46	ClO_4^-	7.6	–1.27
CO_2^{2-}	–4.0	0.40	I^-	8.3	–1.37
SO_4^{2-}	–3.0	0.25	PF_6^-	(12.2)[c]	–1.93[d]
F^-	–2.1	0.11	$B_{12}H_{12}^{2-}$	14.8	–2.31
Cl^-	4.1	–0.76	$B_{12}Cl_{12}^{2-}$	- - -	- - -
Br^-	5.8	–1.01	$B_{12}Br_{12}^{2-}$	- - -	- - -
SCN^-	5.9	–1.03	$B_{12}I_{12}^{2-}$	- - -	- - -

[a] Calculated according to Marcus (see Table S4 for raw data). [b] From eq. 25 in ref. 16, there abbreviated as ΔG_{HB}. [c] Calculated from eq. 25 in ref. 16, by using the reported value for ΔHB. [d] From ref. 15, multiplied with the correction factor of 1.25 subsequently introduced in ref. 16.

Ions unambiguously qualify as water-structure breakers if ΔHB <–1, which is the case for BF_4^-, ClO_4^- I^-, and PF_6^-, ions which conventionally classify as chaotropes in the Hofmeister series. For the borate cluster ion $B_{12}H_{12}^{2-}$ the ΔHB value reaches an extreme value below –2. The ΔS_{struct} and ΔHB values for the larger (perhalogenated) borate clusters cannot be directly calculated due to the lack of hydration entropy data (Table S4). However, from the general correlation of water-structural entropies with ionic radius, it can be projected that their water-breaking properties are even more pronounced than those of the parent. The Marcus model provides therefore theoretical support for the superchaotropic nature of the dodecaborate cluster ions, as revealed by the salting-in experiments (see Section 9).

11. References

1. D. Gabel, D. Moller, S. Harfst, J. Rosler, H. Ketz, *Inorg. Chem.* **1993**, *32*, 2276.
2. I. B. Sivaev, A. B. Bruskin, V. V. Nesterov, M. Y. Antipin, V. I. Bregadze, S. Sjoberg, *Inorg. Chem.* **1999**, *38*, 5887.
3. E. Justus, A. Vöge, D. Gabel, *Eur. J. Inorg. Chem.* **2008**, 5245.
4. CrysAlisPro 2012, Agilent Technologies. Version 1.171.37.31.
5. R. C. Clark, J. S. Reid, *Acta Cryst. A* **1995**, *51*, 887.
6. G. M. Sheldrick, *Acta Cryst. A* **2008**, *64*, 112.
7. O. V. Dolomanov, L. J. Bourhis, R. J. Gildea, J.A.K. Howard, H. Puschmann, *J. Appl Cryst.* **2009**, *42*, 229.
8. A. L. Spek, *Acta Cryst. D* **2009**, *65*, 148.
9. O. Volkov, C. Hu, P. Peatzold, *Z. Anorg. Allg. Chem.* **2005**, *631*, 1107.
10. S. Parsons, H. Flack, *Acta Cryst. A* **2004**, *60*, s61.
11. S. Yoo, Y. A. Lei, X. C. Zeng, *J. Chem. Phys.* **2003**, *119*, 6083.
12. G. Maroulis, D. Xenides, U. Hohm, A. Loose, *J. Chem. Phys.* **2001**, *115*, 7957.
13. D. V. Peryshkov, A. A. Popov, S. H. Strauss, *J. Am. Chem. Soc.* **2009**, *131*, 18393.
14. Y. Marcus, *J. Solution Chem.* **1994**, *23*, 831.
15. Y. Marcus, *Ion Properties, Vol. 1*, Marcel Dekker, New York, **1997**.
16. Y. Marcus, *Chem. Rev.* **2009**, *109*, 1346.
17. Y. Marcus, *Ions in Water and Biophysical Implications. From Chaos to Cosmos*, Springer, London, **2012**.
18. A. Kaczmarczyk, W. C. Nichols, W. Stockmayer, T. B. Eames, *Inorg. Chem.* **1968**, *7*, 1057.
19. It should be noted that the Marcus model is best suited to obtain information on water-structural entropies, while water-structural enthalpies and heat capacities cannot be interpreted in a similarly straightforward manner, see ref. 14 and 17.
